# Dual Projection Fusion for Reference-Based Image Super-Resolution

**DOI:** 10.3390/s22114119

**Published:** 2022-05-28

**Authors:** Ruirong Lin, Nanfeng Xiao

**Affiliations:** School of Computer Science and Engineering, South China University of Technology, Guangzhou 510006, China; xiaonf@scut.edu.cn

**Keywords:** reference-based super-resolution, attention mechanism, texture transformer, dual projection fusion

## Abstract

Reference-based image super-resolution (RefSR) methods have achieved performance superior to that of single image super-resolution (SISR) methods by transferring texture details from an additional high-resolution (HR) reference image to the low-resolution (LR) image. However, existing RefSR methods simply add or concatenate the transferred texture feature with the LR features, which cannot effectively fuse the information of these two independently extracted features. Therefore, this paper proposes a dual projection fusion for reference-based image super-resolution (DPFSR), which enables the network to focus more on the different information between feature sources through inter-residual projection operations, ensuring effective filling of detailed information in the LR feature. Moreover, this paper also proposes a novel backbone called the deep channel attention connection network (DCACN), which is capable of extracting valuable high-frequency components from the LR space to further facilitate the effectiveness of image reconstruction. Experimental results show that we achieve the best peak signal-to-noise ratio (PSNR) and structure similarity (SSIM) performance compared with the state-of-the-art (SOTA) SISR and RefSR methods. Visual results demonstrate that the proposed method in this paper recovers more natural and realistic texture details.

## 1. Introduction

Image super-resolution (SR) aims to reconstruct an HR image with clear texture details from a blurred LR image [1]. In recent years, deep learning-based SISR algorithms [2,3,4,5,6] have made significant progress and are widely used for various real-world tasks, such as medical image processing [7,8], surveillance imaging [9], and object recognition [10]. However, when the upsampling factor reaches 4× or greater, the reconstruction results of most existing methods show blurred visual effects or artifacts. Although generative adversarial network (GAN) [11] and perceptual loss [12]-based methods have been proposed to improve the quality of the reconstructed images, they cannot guarantee the realism of the generated textures, resulting in the degradation of the PSNR performance.

To address this problem, the RefSR method [13,14,15,16,17,18], which transfers fine details from an additional reference image (Ref) to the LR image, is proposed. Compared to traditional SISR, RefSR exhibits better reconstruction performance. RefSR transforms the more complex texture generation process into a relatively simple texture search and transfer operation, thus producing more realistic and natural-looking textures. For example, Zhang et al. [16] feed the Ref and LR images into a pre-trained VGG model for feature extraction, and then performed feature matching and texture transfer in the neural feature space. Yang et al. [18] firstly introduced the transformer architecture to the SR tasks and proposed a novel texture transformer to model the correspondence between the LR and Ref images, which helps to perform feature matching more accurately.

However, the previous methods ignore that the information in the LR space still has valuable high-frequency components. Besides, they simply add or concatenate the LR feature and the Ref feature together without taking the different information between feature sources into account. To tackle the aforementioned issues, we propose a novel RefSR method called DPFSR, which not only makes full use of the high-frequency information from the Ref image and the LR space, but also performs effective feature fusion. In summary, the main contributions of this paper are as follows:

- We propose a lightweight backbone, called deep channel attention connection network (DCACN), which can extract valuable high-frequency components from the LR space for image reconstruction. With the help of DCACN, the proposed DPFSR possesses stronger feature representation capability;
- We also propose a novel fusion module, called dual projection fusion module (DPFM), which enables the network to focus on the different information between feature sources through inter-residual projection operations, generating more discriminative fusion features and further improving the performance of the model;
- We evaluate the proposed DPFSR on three publicly available datasets, and our method proved to be superior to the state-of-the-art SISR and RefSR methods through quantitative and qualitative comparisons. Furthermore, we also conduct an ablation study to explore the effect of utilizing reference images with different similarity levels on the model performance. Experimental results demonstrate that the proposed approach possesses superior robustness.

The rest of this paper is organized as follows. In Section 2, we review some deep learning-based SISR and RefSR approaches. In Section 3, we discuss the proposed network framework. Section 4 discusses the details of the experiments and the analysis of the results. Finally, the summary of this paper is given in Section 5.

## 2. Related Work

### 2.1. Single Image Super-Resolution

Deep learning-based SISR algorithms have attracted significant attention in recent years. Dong et al. first applied convolutional neural networks (CNNs) to image SR reconstruction and proposed SRCNN [2]. Later, Dong et al. proposed a faster and more efficient FSRCNN [19] model based on SRCNN, which directly takes the LR image as input and then adopts deconvolution at the end of the network to upscale the feature maps, greatly reducing the number of network parameters. Kim et al. introduced residual learning structures and recurrent neural networks into the optimization process of the network and proposed VDSR [20] and DRCN [21], which can effectively control the number of parameters while increasing the depth of the network. To improve the processing speed of the network, Shi et al. reconstructed the HR image using sub-pixel convolution [22] rather than deconvolution. Lai et al. [23] combined the idea of progressive image reconstruction to super-resolve the LR image in a step-by-step zooming manner. Lim et al. [5] removed the BN layer in the residual network, thus building a deeper SR network. Moreover, Zhang et al. combined the dense structure and residual structure to construct a RDN [24], resulting in faster convergence of the network. Recently, RCAN [25] adopted the residual-in-residual (RIR) architecture and introduced the channel attention mechanism to achieve superior PSNR performance.

The methods mentioned above mainly focus on minimizing MAE or MSE loss, giving the reconstructed images a high PSNR value. However, they often fail to recover texture details of the image effectively and are thus unsatisfactory in terms of perceptual quality. For this reason, Johnson et al. [12] proposed a perceptual-driven SR model that minimizes the distance between the semantic features extracted from the VGG network to improve visual quality. Inspired by GAN, Ledig et al. proposed SRGAN [26], which utilizes content loss and adversarial loss as the objective function for optimization, yielding more natural image texture. Later, Wang et al. [27] improved on the structure of SRGAN to further enhance the image reconstruction performance. Zhang et al. [28] trained a ranker that can simulate perceptual metrics. Moreover, they introduced rank-content loss to produce visually more plausible results. Recently, Ma et al. [29] introduced the notion of gradient guidance to super-resolution tasks, which retains the advantages of GAN while reducing the image distortion problem to achieve state-of-the-art perceptual results.

### 2.2. Reference-Based Image Super-Resolution

Compared with SISR, which only involves a single LR image as input, RefSR super-solves the LR image by leveraging the high-frequency details provided by an additional Ref image with similar content. RefSR transforms more complex texture generation into relatively simple texture search and transfer, effectively improving the performance of SR reconstruction. The key factor that affects the performance of RefSR is how to transfer suitable high-frequency texture details from the Ref image as auxiliary information for image reconstruction, which is generally performed in two ways, namely pixel-wise alignment and patch-wise matching.

One solution of RefSR is to perform pixel-wise alignment between the LR and Ref images. Specifically, Yue et al. [30] achieved the goal of aligning the LR and Ref images by a global registration operation, followed by a local matching operation to super-resolve the LR image. Zheng et al. proposed a RefSR model called CrossNet [15], which aligns the Ref and LR images using optical flow and warps features at different scales according to the flow. However, CrossNet suffers severe performance degradation when there are large displacements between the Ref and LR images. To alleviate this issue, Shim et al. [31] employed deformable convolution [32] rather than optical flow to estimate the offset between the Ref and LR images, which effectively improves image alignment quality. Nevertheless, these methods have limitations in capturing long-range dependencies.

Another solution adopts the patch-wise matching scheme to search for related texture features from the Ref image, which is not limited by long-range dependencies and thus is more flexible. Zheng et al. [14] defined RefSR as a two-stage task, where patch matching is performed in the first stage to find feature correspondence, and feature synthesis is performed according to the correspondence in the second stage. SRNTT [16] fed the Ref and LR images into a pre-trained VGG network for feature extraction, then applied dense patch matching to calculate texture similarity between the Ref and LR feature, and used it to adaptively transfer high-frequency details from the Ref image into the LR image. Yue et al. used a learnable texture extractor instead of a fixed VGG network for feature extraction, and the proposed TTSR [18] is capable of achieving more accurate patch matching and texture feature transfer. However, the above methods, such as SRNTT and TTSR, ignore the fact that the LR space still contains valuable high-frequency details. Besides, they simply add or concatenate the LR and transferred Ref features together, which cannot effectively fuse these two independently extracted features. In this paper, we propose a novel backbone for extracting more elaborate features from the LR space, as well as a new fusion module for combining the LR and Ref features more efficiently.

## 3. Methods

**Figure 1:**
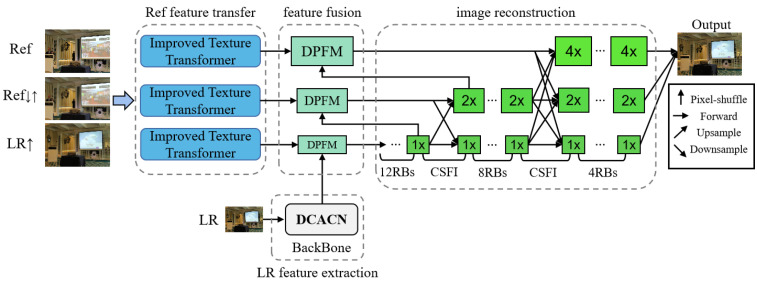
The overall structure of the proposed dual projection fusion for reference-based image super-resolution (DPFSR).

The overall structure of the proposed dual projection fusion for reference-based image super-resolution (DPFSR) is shown in Figure 1. The proposed DPFSR can be divided into four parts: LR feature extraction with the deep channel attention connection network (Section 3.1), Ref feature transfer with the improved texture transformer (Section 3.2), feature fusion with the dual projection fusion module (Section 3.3), and image reconstruction with the cross-scale feature integration module (Section 3.4).

Let us denote $I^{LR}$ and $I^{SR}$ as the input and output of DPFSR. $I^{Ref}$, $I^{Ref↓↑}$, and $I^{LR↑}$ denote the reference image, the 4× bicubic-downsampled and upsampled reference image, and the 4× bicubic-upsampled input image, respectively. We first adopt a backbone network to extract the feature $F_{LR}$ from the LR input:

(1) $$F_{LR}=H_{DCACN}\left(I^{LR}\right)$$

where $H_{DCACN}\left(\cdot \right)$ represents our proposed deep channel attention connection network (DCACN). Then, taking $I^{Ref}$, $I^{Ref↓↑}$, and $I^{LR↑}$ as inputs, the texture feature $F_{Ref}$ is transferred from the reference image using the improved texture transformer (ITT):

(2) $$F_{Ref}=H_{ITT}\left(I^{Ref},I^{Ref↓↑},I^{LR↑}\right)$$

where $H_{ITT}\left(\cdot \right)$ denotes the output of ITT. Note that ITT can be further stacked to transfer texture features of different scales. $F_{LR}$ and $F_{Ref}$ are then used for feature fusion with the dual projection fusion module (DPFM):

(3) $$F_{fused}=H_{DPFM}\left(F_{LR},F_{Ref}\right)$$

where $F_{fused}$ represents the fused comprehensive feature. Finally, the fused feature is used for image reconstruction:

(4) $$I^{SR}=H_{REC}\left(F_{fused}\right)$$

where $H_{REC}\left(\cdot \right)$ denotes the reconstructed network with the cross-scale feature integration module (CSFI).

The objective functions used to train the network will be described in detail in Section 3.5.

### 3.1. Deep Channel Attention Connection Network

**Figure 2:**
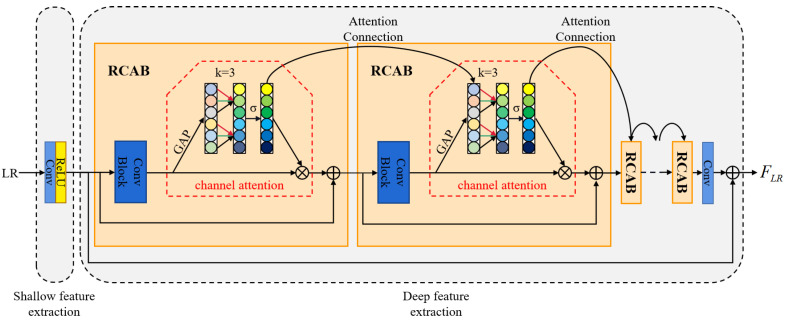
An illustration of the deep channel attention connection network (DCACN). The ConvBlock in RCAB consists of two convolutional layers and a ReLU activation layer placed between them.

Inspired by [33,34], we propose a deep channel attention connection network (DCACN) to exploit the valuable high-frequency information in LR space. The proposed DCACN makes full use of the interdependence among the channel-wise features and the information flow among attention blocks to build a solid foundation for image reconstruction. As shown in Figure 2, the proposed DCACN mainly consists of two parts: a shallow feature extraction and a deep feature extraction part [35]. We use a convolutional layer and a ReLU [36] activation layer to extract the shallow features of the input image, while the deep feature extraction part consists of multiple residual channel attention blocks (RCABs) with connected attention (CA), a convolutional layer, and a long skip connection (LSC). Since the shallow feature extraction part we use is the same as that in previous works [16,18], we pay more attention to the deep feature extraction. More details about RCAB and CA are given as follows.

For a given feature map $F\in R^{H\times W\times C}$ produced by a convolutional block, we adopt the global averaging pooling (GAP) operation to generate the channel statistics as $E\in R^{1\times 1\times C}$, which is obtained by shrinking *F* through spatial dimensions H×W [37]:

(5) $$E=H_{GAP}\left(F\right)=\frac{1}{H\times W}\sum_{i=1}^{H}\sum_{j=1}^{W}F\left(i,j\right)$$

where $H_{GAP}\left(\cdot \right)$ represents the global averaging pooling operation; F(i,j) is the value at position (i,j) of *F*.

After that, we perform a faster 1D convolution followed by a gating mechanism with sigmoid [38] activation to generate the attention map *S* [33]:

(6) $$S=\sigma (Conv1D_{3}\left(E\right))$$

where $Conv1D_{3}\left(\cdot \right)$ indicates 1D convolution with kernel size of 3. As explained in [34], attention maps are essential for attention learning, and the addition of an attention connection mechanism can further facilitate information flow among attention blocks. Consequently, we incorporate the informative features of the previous attention block into the current attention block by applying the attention connection mechanism. The resulting attention map can be represented as [34]:

(7) $$S=\sigma (Conv1D_{3}\left(\alpha E+\beta \tilde{S}\right))$$

where α and β are learnable parameters. For the first residual channel attention block in the deep feature extraction part, (α, β) is set to (1, 0); then, Equation (7) is simplified to Equation (6). $\tilde{S}$ is the attention map produced by the previous attention block. Finally, *S* is used to rescale *F*:

(8) $$F^{\prime }=F⊗S$$

where ⊗ represents element-wise multiplication.

In summary, the proposed DCACN can effectively capture cross-channel interactions for channel attention learning, thus facilitating the mining of valuable high-frequency information from the LR features.

### 3.2. Improved Texture Transformer

**Figure 3:**
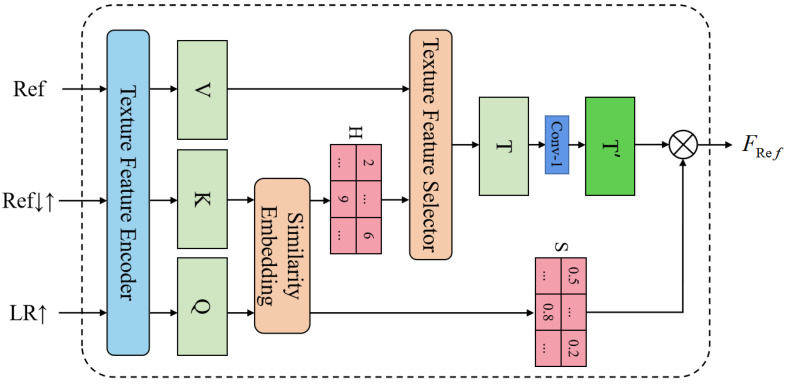
The improved texture transformer (ITT).

As shown in Figure 3, the proposed ITT mainly consists of three parts: the texture feature encoder (TFE), the similarity embedding module (SE), and the texture feature selector (TFS). LR↑ and Ref denote the 4× bicubic-upsampled LR and Ref images, respectively. Ref↓↑ is obtained by sequentially applying 4× bicubic-downsampling and upsampling to Ref. By doing so, it is ensured that Ref↓↑ matches the frequency domain of LR↑. After that, ITT takes LR↑, Ref↓↑, and Ref as inputs and outputs a reference texture feature. Details will be discussed below.

#### 3.2.1. Texture Feature Encoder

We use the first 12 layers of the VGG19 [39] network as the texture feature encoder, which can extract three different scales of feature maps (4×, 2×, 1×) from the relu1_2, relu2_1, and relu3_1 layers of the VGG19 network. Based on the properties of TFE, we can stack multiple ITTs and perform similarity embedding at different extraction scales to transfer multi-scale texture features. More details will be discussed in Section 3.4. The encoding process is defined as [18]:

(9) Q=TFE(LR↑)

(10) K=TFE(Ref↓↑)

(11) V=TFE(Ref)

where TFE(·) denotes the output of the texture feature encoder. Note that the parameters of the texture feature encoder are first initialized with the pre-trained model of VGG19, after which the encoder will continue training along with the whole network. The output features *Q*, *K*, and *V* will be further processed in the next modules.

#### 3.2.2. Similarity Embedding Module

The similarity embedding module (SE) aims to find the correspondence between the Ref and LR images. To this end, *Q* is unfolded into $H_{LR}\times W_{LR}$ patches $\left\{q_{1},\ldots ,q_{H_{LR}\times W_{LR}}\right\}$, and *K* is unfolded into $H_{Ref}\times W_{Ref}$ patches $\left\{k_{1},\ldots ,k_{H_{Ref}\times W_{Ref}}\right\}$. Then, for each patch of *Q*, we find its most relevant patch in *K*. Specifically, we perform the dense patch matching on the unfolded patches of *Q* and *K*. Taking the *i*-th patch $q_{i}$, for example, we compute the cosine similarity [40] with each patch of *K* as:

(12) $$r_{i,j}=\langle \frac{q_{i}}{∥q_{i}∥},\frac{k_{j}}{∥k_{j}∥}\rangle$$

where $q_{i}$ is the *i*-th patch of *Q*, $k_{j}$ is the *j*-th patch of *K*, and $r_{i,j}$ is their similarity score. Next, we utilize $r_{i,j}$ to calculate the hard-attention map *H* and the soft-attention map *S*. The *i*-th position of *H* is calculated as:

(13) $$h_{i}=\underset{j}{arg\;max}\;r_{i,j}$$

The *i*-th position of *S* is the highest similarity score associated with the *i*-th patch $q_{i}$ in *Q*, which is computed as:

(14) $$s_{i}=\underset{j}{max}\;r_{i,j}$$

#### 3.2.3. Texture Feature Selector

The texture feature selector aims to transfer high-resolution texture features from the Ref image. To this end, we extract related patches from *V* with the guidance of the hard attention map *H* as [18]:

(15) $$t_{i}=v_{h_{i}}$$

where $t_{i}$ is the *i*-th element of *T*. We fold these extracted patches to form the feature map *T*. To save the computational cost of the network, we perform a 1×1 convolutional layer on *T* to decrease the number of feature map channels, and obtain a new feature map $T^{\prime }$:

(16) $$T^{\prime }=Conv\left(T\right)$$

In addition, we multiply $T^{\prime }$ element-wise with the soft attention map *S* to obtain the final reference texture features. By doing so, the transferred texture features with high-correlation will be enhanced, while low-correlation ones will be suppressed [35]. This process can be expressed as:

(17) $$F_{Ref}=T^{\prime }⊗S$$

where $F_{Ref}$ represents the high-resolution texture features transferred by ITT. ⊗ denotes the element-wise product operation.

Compared with the texture transformer (TT) proposed in [18], ITT uses deeper features for similarity embedding so that it can transfer more accurate texture features from Ref. In addition, it also uses a 1 × 1 convolutional layer for data dimensionality reduction of the preliminary extracted texture feature T. Such a design allows ITT to effectively reduce the number of module parameters while maintaining considerable performance.

### 3.3. Dual Projection Fusion Module

**Figure 4:**
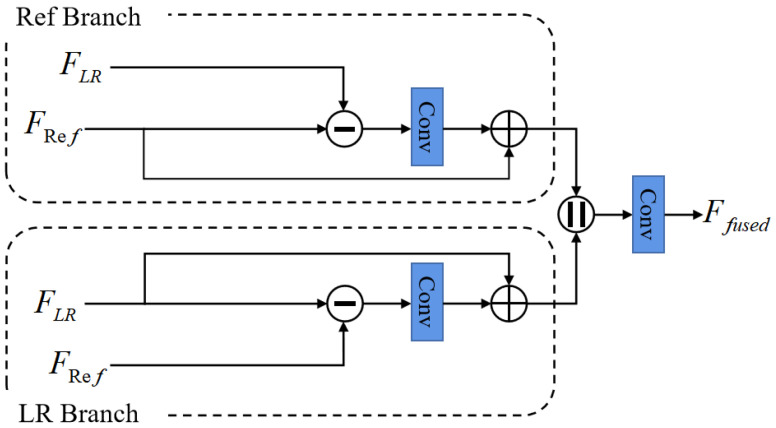
An illustration of the proposed dual projection fusion module (DPFM).

Since the LR features $F_{LR}$ and the transferred texture features $F_{Ref}$ originate from different information sources, the question of how to fuse them into a comprehensive feature map is essential to synthesizing the final super-resolve results [41]. Unlike previous works that simply add or concatenate them together, we propose a dual projection fusion module (DPFM) to effectively combine them together, as shown in Figure 4.

Our DPFM consists of two branches: the Ref branch and the LR branch. Such a design allows the network to further refine the information that differ from each other. Taking the Ref branch as an example, we first compute the residual between $F_{Ref}$ and the LR features $F_{LR}$, then apply a convolutional layer with kernel size of 3 to the residual and add it back to the $F_{Ref}$ to obtain a more discriminative feature representation $F_{Ref}^{\prime }$:

(18) $$F_{Ref}^{\prime }=F_{Ref}+Conv\left(F_{Ref}-F_{LR}\right)$$

Similarly, the processing procedure of the LR branch can be expressed as:

(19) $$F_{LR}^{\prime }=F_{LR}+Conv\left(F_{LR}-F_{Ref}\right)$$

After that, we concatenate the output features from two branches and apply a 3×3 convolution layer to obtain the final fused features:

(20) $$F_{fused}=Conv\left(Concat\left(F_{LR}^{\prime },F_{Ref}^{\prime }\right)\right)$$

where Concat indicates the concatenation operation along the channel dimension.

In summary, the proposed DPFM fully considers the differences between the LR features and the transferred texture features, and then fuses them into a more representative and comprehensive feature representation, laying a solid foundation for the next stage of image reconstruction.

### 3.4. Image Reconstruction

Compared with single-scale features, multi-scale features contain richer information, which is beneficial to enhancing the effect of image super-resolution reconstruction. Therefore, we stack multiple ITTs to gradually fuse the multi-scale Ref features (1×,2× and 4×) into the LR features. Moreover, we apply the CSFI module [18] to facilitate the feature exchange across each scale whenever upsampling the LR feature to the next scale. In this way, we can fully fuse the texture feature information at different scales, thus achieving a more powerful feature representation capability. The reconstruction procedure is shown in the right half of Figure 1.

### 3.5. Loss Function

As in [18], the loss function used in this paper contains three losses. The overall loss function is defined as follows:

(21) $$L=\lambda_{rec}L_{rec}+\lambda_{per}L_{per}+\lambda_{adv}L_{adv}$$

- Reconstruction loss: $L_{rec}$ is the Mean absolute error (MAE) loss:
  (22) $$L_{rec}={∥I^{HR}-I^{SR}∥}_{1}$$
  where $I^{HR}$ and $I^{SR}$ represent the ground-truth and the output of our network;
- Perceptual loss: Perceptual loss $L_{per}$ aims to improve the visual quality of the recovered image. In this paper we uses the conventional perceptual loss [12]:
  (23) $$L_{per}={∥\phi_{relu5\_1}^{vgg}\left(I^{HR}\right)-\phi_{relu5\_1}^{vgg}\left(I^{SR}\right)∥}_{2}^{2}$$
  where $\phi_{relu5\_1}^{vgg}\left(\cdot \right)$ denotes the relu5_1 layer output features of the VGG19 model;
- Adversarial loss: $L_{adv}$ is the adversarial loss that promotes the synthesized images to obtain clear and natural image details. Here, we also adopt the WGAN-GP [42]:
  (24) $$L_{D}=\underset{\tilde{x}∼P_{g}}{E}\left[D\left(\tilde{x}\right)\right]-\underset{x∼P_{r}}{E}\left[D\left(x\right)\right]+\lambda \underset{\hat{x}∼P_{\hat{x}}}{E}[(∥\nabla_{\hat{x}}D\left(\hat{x}\right){∥}_{2}-1{)}^{2}]$$
  (25) $$L_{G}=-\underset{\tilde{x}∼P_{g}}{E}\left[D\left(\tilde{x}\right)\right]$$
  where $L_{D}$ and $L_{G}$ denote the discriminator loss and generator loss in WGAN-GP, respectively. Note that the generator refers to DPFSR in this paper. *D* is the set of 1-Lipschitz functions. $\tilde{x}$ and *x* denote the output of the generator and the real data, respectively. $\hat{x}$ is defined as the data randomly sampled along the line between $\tilde{x}$ and *x*, and $P_{\hat{x}}$, $P_{g}$, and $P_{r}$ are the data distributions they obey.

## 4. Experiments and Results

### 4.1. Datasets and Evaluation Metrics

We followed previous work [18] to train our model using the training set of the CUFED5 dataset, which is collected from photo albums depicting daily life events and contains a total of 11,871 image pairs. Each image pair has a ground-truth image and a corresponding reference image. To verify the robustness of the model, we evaluated it on three publicly available datasets: the testing set of the CUFED5 dataset, the Sun80 [43] dataset, and the Urban100 [44] dataset. The CUFED5 testing set contains 126 sets of images, each with one high-resolution input image and five reference images with different similarity levels. The Sun80 dataset contains 80 images from natural scenes; each image is paired with several references. The Urban100 dataset consists of 100 images of indoor, urban, and architectural scenes with strong self-similarity. Due to its lack of reference images, we use its LR images instead. All the SR results are measured on Y channel of the YCrCb color space by PSNR and SSIM [45] metrics.

### 4.2. Implementation Details

We construct LR images by performing 4× bicubic interpolation downsampling on HR images in all experiments. The number of RCAB in DCACN is set to 20. The dense patch matching operation is performed only on the smallest scale (1×) in the TFE and propagates the correspondence to other scales (2×, 4×). During training, training examples are augmented by randomly rotating 90°, 180°, 270°, and randomly flipping horizontally and vertically. Our network was trained with a batch size of nine, and an Adam [46] optimizer with $\beta_{1}$ = 0.9 and $\beta_{2}$ = 0.999 was used to optimize the network. The learning rates of both the generator and the discriminator were set to $1e^{-4}$. The weights for $L_{rec}$, $L_{per}$, and $L_{adv}$ were 1, $1e^{-2}$, and $1e^{-3}$, respectively. We initially pre-trained the network with only 10 epochs using the reconstruction loss, then fine-tuned the network by applying all losses and continued training for 60 epochs. The proposed DPFSR was implemented on a NVIDIA 1080 Ti GPU using the PyTorch [47] framework; more information on the experimental configuration is shown in Table 1.

### 4.3. Ablation Study

In this section, we conduct ablation studies to investigate the effectiveness of several important designs in our network. Furthermore, we also investigate the effect of using reference images with different similarity levels on model performance.

#### 4.3.1. Effect of DPFM and DCACN

The dual projection fusion module DPFM is used to fuse the LR features extracted by DCACN with the transferred Ref features. To independently verify the effectiveness of DPFM, we first removed the attention mechanism and connected attention in DCACN and then replace DPFM with a concatenation operation and a convolutional layer to construct the “Base” model. As illustrated in Table 2, we can observe that the PSNR performance is not greatly improved if only one branch in the DPFM is used. Taking “Base + LR branch of DPFM” as an example, while the inter-residual projection operation enables the LR branch to obtain useful high-frequency information from $F_{Ref}$, it also loses the rich common information in the Ref branch. If the complete DPFM is used, the PSNR value will be improved from 27.18 dB to 27.23 dB compared with the “Base” model. Furthermore, to verify the effectiveness of DCACN, we continued to add the attention mechanism and connected attention to the “Base + DPFM” model. With the help of attention mechanism and connected attention, we found that the constructed “DPFSR” model achieves a performance gain of 0.02 dB over the “Base + DPFM” model.

#### 4.3.2. Effect of ITT

Table 3 provides the ablation results on improved texture transformer. We first replaced the ITT with TT to construct the “DPFSR (replace ITT with TT)” model. It is worth noting that in TT, the LR features are simply concatenated with the transferred texture features and then fused together by a convolution layer, which means that this model does not use DPFM. For fair comparison, we constructed another comparison model, “DPFSR (use ITT without DPFM)”, by removing the DPFM and using a concatenation operation and a convolution layer instead. With the ITT, we can find that the PSNR value increases by 0.02 dB. This result shows that the ITT is capable of transferring more accurate texture features from Ref. On the other hand, it also demonstrates that the combination of ITT and DCACN can achieve better performance.

#### 4.3.3. Effect of Different Reference Similarity Levels

We ran ablation experiments on the CUFED5 to investigate the effect of reference images with different similarity levels on the model performance. In Table 4, the similarity level from “L1” to “LR” gradually decreases. Among them, “LR” denotes utilizing the low-resolution image itself as the reference. We can observe that the performance of the model benefits from the similarity level between the reference and LR image. The higher the similarity, the better the performance. Compared with the previous state-of-the-art RefSR methods, we achieved the best performance at every similarity level. Especially when using “LR” as the reference image, the proposed DPFSR-*rec* can improve the performance of PSNR by 0.2 dB compared to TTSR-*rec*.

### 4.4. Comparisons with State-of-the-Art Methods

We made a comparison of the proposed DPFSR with various SR methods, including state-of-the-art SISR methods and RefSR methods. For SISR methods, we included SRCNN [2], MDSR [5], RDN [24], RCAN [25], SRGAN [26], ENET [48], ESRGAN [27], RSRGAN [28], and SPSR [29]. For RefSR methods, CrossNet [15], SRNTT [16], and TTSR [18] were compared with our method. To achieve fair comparison, all methods were trained using the training set of the CUFED5 dataset and were tested on the CUFED5, Sun80, and Urban100 datasets.

#### 4.4.1. Quantitative Evaluation

Table 5 reports the quantitative comparison results on the three testing benchmarks. The best results are highlighted in red, while the second-best results are highlighted in blue. Note that the RefSR method with the suffix “-*rec*” indicates training with reconstruction loss only, aiming to achieve a higher PSNR. It can be found that our method achieves the state-of-the-art PSNR performance for all the testing benchmarks. On the CUFED5 testing set, the proposed method outperforms the state-of-the-art method by 0.16 dB. Moreover, we achieve 0.08 dB and 0.16 dB improvements on the Sun80 and Urban100 datasets, respectively. The superior performance demonstrates the effectiveness of our well-designed module.

In addition, we also considered the comparison with other patch-based RefSR methods in the number of network parameters and execution time. We took five pairs of images as input to calculate the average execution time, where each pair contains a 120 × 120 LR image and a 400 × 400 Ref image, respectively. As described in Table 6, it can be found that TTSR and DPFSR are significantly faster than SRNTT in terms of execution time. Combining the results in Table 5, we can conclude that DPFSR achieves the best performance on PSNR and SSIM, although it is slightly inferior to TTSR in terms of the number of network parameters and execution time.

#### 4.4.2. Qualitative Evaluation

**Figure 5:**
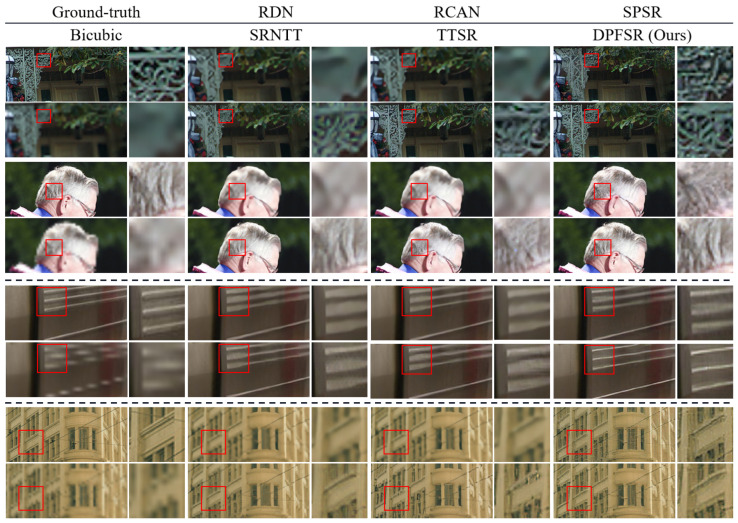
Qualitative comparison with other SR methods on the CUFED5 testing set (the top two examples), Sun80 [43] (the third example), and Urban100 [44] (the bottom example). To see the details of the image clearly, the area marked by the red frame is enlarged.

Figure 5 shows the results of the qualitative comparison. We can observe that the SISR methods suffer from the distinct blurring artifacts because it only utilizes the information from the LR image. In contrast, our method shows visual results with more natural and realistic textures. In addition, our method also has greater relevancy to the ground-truth images than other RefSR methods. The qualitative comparison indicates that our proposed DPFSR can effectively fuse the transferred texture features into the LR features, which is beneficial for generating satisfactory SR results. Therefore, the proposed DPFSR is perceptually superior to other state-of-the-art methods.

## 5. Conclusions

In this paper, we propose a dual projection fusion for reference-based image super-resolution (DPFSR). The proposed dual projection fusion module can effectively combine the transferred texture feature and the LR feature to form more discriminative fusion feature maps. Moreover, we propose a novel backbone termed deep channel attention connection network, which is capable of extracting valuable high frequency components from the LR space. Such a design allows the proposed DPFSR to recover images with more natural and realistic texture details. Experimental results show that our DPFSR achieves the best performance in terms of both quantitative and qualitative comparisons. Specifically, DPFSR achieves PSNR gains of 0.16 dB, 0.08 dB, and 0.16 dB on three benchmark datasets (CUFED5, Sun80, and Urban100) compared to TTSR. In the future, we would like to explore the application of the RefSR in real-world scenarios such as medical imaging and dual-camera imaging. Furthermore, we will also try to stack more ITTs to achieve super-resolution at larger scale factors (8×, 16×) and try to handle the input data in the presence of noise.

## Figures and Tables

**Table 1:** Experimental configuration.

Experimental Configuration	Options
Linux version	Ubuntu 20.04
Deep-learning framework	PyTorch 1.10
CUDA version	11.2
Input patchsize	160 × 160
Reference patchsize	160 × 160
Scale factor	4×

**Table 2:** Ablation study on the dual projection fusion module and deep channel attention connection network.

Model	PSNR/SSIM
Base	27.18/0.806
Base + LR branch of DPFM	27.18/0.807
Base + Ref branch of DPFM	27.16/0.805
Base + DPFM	27.23/0.807
DPFSR (Ours)	27.25/0.808

**Table 3:** Ablation study on improved texture transformer.

Model	PSNR/SSIM
DPFSR (replace ITT with TT)	27.18/0.806
DPFSR (use ITT without DPFM)	27.20/0.807

**Table 4:** Ablation study on reference images with five different similarity levels.

Level	CrossNet	SRNTT-*rec*	TTSR-*rec*	DPFSR-*rec*
	PSNR/SSIM	PSNR/SSIM	PSNR/SSIM	PSNR/SSIM
L1	25.48/0.764	26.15/0.781	26.99/0.800	27.15/0.805
L2	25.48/0.764	26.04/0.776	26.74/0.791	26.86/0.794
L3	25.47/0.763	25.98/0.775	26.64/0.788	26.73/0.790
L4	25.46/0.763	25.95/0.774	26.58/0.787	26.68/0.789
LR	25.46/0.763	25.91/0.776	26.43/0.782	26.63/0.786

**Table 5:** Quantitative comparison with other SR methods on three benchmark datasets. We group methods by SISR (top) and RefSR (bottom). The best performance is highlighted in red, while the second-best performance is highlighted in blue.

Method	CUFED5	Sun80	Urban100
	PSNR/SSIM	PSNR/SSIM	PSNR/SSIM
Bicubic	24.22/0.684	28.65/0.766	23.13/0.659
SRCNN [2]	25.33/0.745	28.26/0.781	24.41/0.738
MDSR [5]	25.93/0.777	28.52/0.792	25.51/0.783
RDN [24]	26.17/0.771	29.97/0.812	25.59/0.768
RCAN [25]	26.19/0.771	30.02/0.813	25.65/0.771
SRGAN [26]	24.40/0.702	26.76/0.725	24.07/0.729
ENet [48]	24.24/0.695	26.24/0.702	23.63/0.711
ESRGAN [27]	21.90/0.633	24.18/0.651	20.91/0.620
RSRGAN [28]	22.31/0.635	25.60/0.667	21.47/0.624
SPSR [29]	24.39/0.714	27.94/0.744	24.29/0.729
CrossNet [15]	25.48/0.764	28.52/0.793	25.11/0.764
SRNTT-*rec* [16]	26.24/0.784	28.54/0.793	25.50/0.783
SRNTT [16]	25.61/0.764	27.59/0.756	25.09/0.774
TTSR-*rec* [18]	27.09/0.804	30.02/0.814	25.87/0.784
TTSR [18]	25.53/0.765	28.59/0.774	24.62/0.747
DPFSR-*rec*	27.25/0.808	30.10/0.815	26.03/0.787
DPFSR	25.23/0.749	28.42/0.762	24.35/0.734

**Table 6:** Comparison of the number of network parameters and execution time. The methods used for comparison are all patch-based RefSR methods.

Method	Param. (M)	Average Execution Time (ms)
SRNTT [16]	5.74	3811.18
TTSR [18]	6.73	198.58
DPFSR	6.91	212.19

## Data Availability

Publicly available datasets were analyzed in this study. Our training set CUFED5 can be obtained from: https://drive.google.com/drive/folders/1hGHy36XcmSZ1LtARWmGL5OK1IUdWJi3I (accessed on 20 April 2022). The test sets Sun80 and Urban100 are available online at: https://github.com/jbhuang0604/SelfExSR (accessed on 20 April 2022).
